# Biomechanical considerations for an easily-restricted robot-assisted kinematic alignment: a surgical technique note

**DOI:** 10.1186/s42836-023-00191-6

**Published:** 2023-06-05

**Authors:** Pieralberto Valpiana, Stefano Ghirardelli, Rosa Susanna Valtanen, Salvatore Risitano, Ferdinando Iannotti, Christian Schaller, Karlos Zepeda, Michael Engl, Pier Francesco Indelli

**Affiliations:** 1 Südtiroler Sanitätsbetrieb, 39042 Brixen, Italy; 2 Institute for Biomedicine, EURAC Institute, 39100 Bozen, Italy; 3 Personalized Arthroplasty Society (PAS), One Glenlake Parkway NE, Suite 1200, Atlanta, GA 30328 USA; 4 Institute of Biomechanics, Paracelsus Medical University, 5020 Salzburg, Austria; 5 Department of Orthopaedic Surgery, Stanford University School of Medicine, Redwood City, Stanford, CA 94063 USA; 6 Department of Orthopaedics and Traumatology, University of Turin, CTO, 10126 Turin, Italy; 7 Division Orthopaedic Surgery, Ospedale San Paolo, 00053 Civitavecchia, Italy; 8 Touro College of Osteopathic Medicine, New York, NY 10027 USA

**Keywords:** Total knee arthroplasty (TKA), Kinematic alignment (KA), Restricted kinematic alignment (rKA), Robotics, Knee, Alignment

## Abstract

**Background:**

In total knee arthroplasty, the normal kinematics of the knee may not be restored solely based on preoperative gait, fluoroscopic-based, and dynamic radiostereometric analyses.

**Surgical technique case presentation:**

This note introduced a 69-year-old male patient who sustained post-traumatic osteoarthritis of his right knee. He underwent robot-assisted total knee arthroplasty based on anatomical reproduction of knee stability during the swing phase of gait. The kinematic alignment was simply achieved within an easy-to-identified range after preoperative radiographic assessment, intraoperative landmarking and pre-validated osteotomy, and intraoperative range of motion testing.

**Conclusions:**

This novel technique allows personalized and imageless total knee arthroplasty. It provides a preliminary path in reproducing the anatomy alignment, natural collateral ligament laxity, and accurate component placement within safe-to-identified alignment boundaries.

## Introduction

Total knee arthroplasty (TKA) is a successful procedure for almost four decades. Despite multiple innovations in implant design, normal kinematics of the knee is rarely reproduced solely based on preoperative gait, fluoroscopic, and dynamic radiostereometric analyses [1–4].

One of the most cited reasons for this failure has been the use of a mechanical alignment surgical technique; historically, during alignment of the lower extremity, surgeons used this technique, which routinely alters preoperative knee anatomy and constitutional limb alignment, joint line obliquity, joint line distance from the femoral epicondyles, and overall soft tissue tension. As a result, this has prompted the development of alternative alignment techniques that aim to restore more natural knee kinematics. In searching for the perfect alternative to standard alignment, kinematic alignment (KA) [5], restricted kinematic alignment (rKA) [6], personalized alignment [7], restricted inverse kinematic alignment [8], functional alignment [9], and other alignment techniques have been proposed in rapid succession over the last ten years. While they have shown promise in achieving more natural knee kinematics, many of them require complex and time-consuming preoperative planning and were difficult to reproduce intraoperatively.

Currently, the reproduction of constitutional knee alignment can improve using robot-assisted systems, including patient-specific cutting guides, three-dimensional planning software, intraoperative navigation systems, and active-, semi-active, and passive robots. Among them, the ROSA Knee System (Zimmer Biomet, Warsaw, IN, USA) represents a semi-active robotic system allowing cutting jigs according to preoperative deformity, soft tissue tension, and intraoperative imageless planning. The surgeons can estimate real-time extension, mid-flexion, and full flexion gaps to facilitate bone cutting. Previous studies have demonstrated the accuracy of this system regarding resection depth, lateral distal femoral angle (LDFA), and medial proximal tibial angle (MPTA) planning reproduction, implant positioning, and finally, patient-reported outcome scores [10].

This note introduced a novel, robot-assisted technique to reproduce nearly normal kinematics in total knee arthroplasty. The constitutional knee anatomy was reproduced within an easy-to-identified and safe alignment range.

## Surgical technique establishment

A 69-year-old male patient sustained a complete anterior cruciate ligament injury in the right knee 15 years prior to presentation to our clinic. The ligament was reconstructed with a conventional single-tunnel technique. Unfortunately, he gradually developed right knee pain and stiffness due to the development of severe knee osteoarthritis. The patient was admitted to our hospital in December 2022 to undergo total knee arthroplasty surgery. On physical assessment, the patient’s BMI was 31 kg/m^2^. The patient was able to have an antalgic and slightly stiff gait. There was slight atrophy of his right quadriceps. Tenderness was found at the medial and lateral knee compartments but not at the patellofemoral compartment. The knee had a varus deformity on weight-bearing standing films, whereas the varus stress test, valgus stress test and patellar compression test were all negative. The visual analogue scale (VAS) score for his knee pain was 8/10: active ROM was 8° to 110°.

**Fig. 1:**
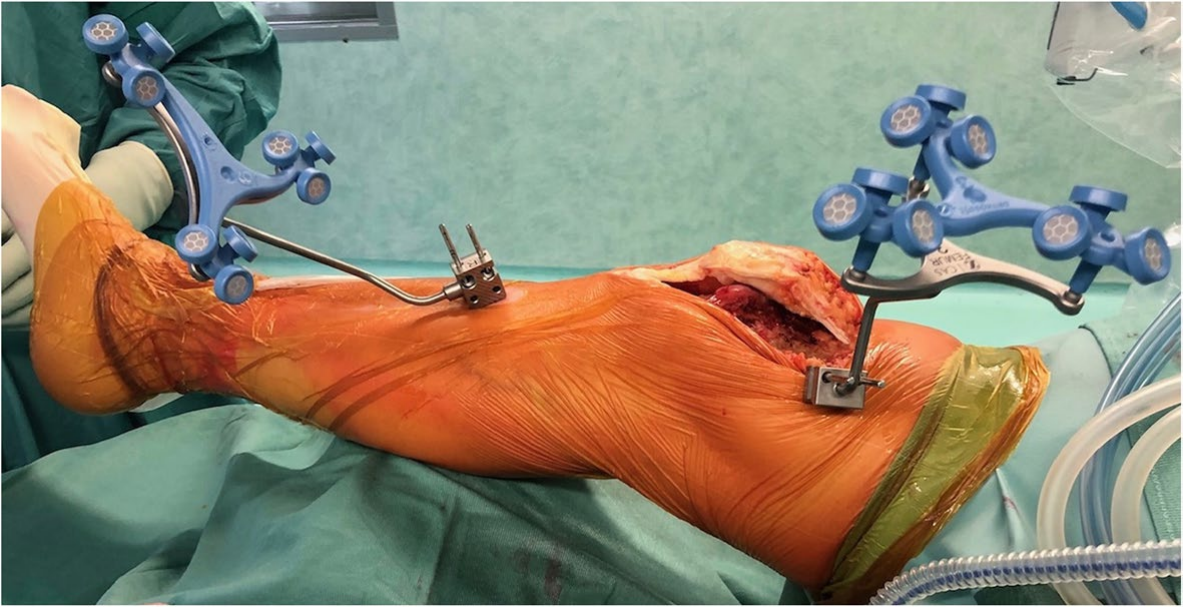
Right knee. Intra-articular positioning of the femoral tracker and extra-articular positioning of the tibial tracker

The patient was scheduled to undergo robot-assisted TKA according to the restricted kinematic alignment (rKA) principles. The operation was performed through the standard medial approach. Medial parapatellar capsulotomy was performed to expose the medial distal femur, where a femoral tracker was intra-articularly positioned. The recommended position was 5 cm proximal to the medial femoral epicondyle, 45° inclination concerning the bone to enhance tracker reading (Fig. 1). The pins for the tibial tracker were positioned on the tibia, 5 cm distal from the tibial tuberosity. Removal of osteophytes was not required at this phase.

The ROSA registration pointer (Zimmer Biomet, Warsaw, IN, USA) was then used to determine the following femoral and tibial landmarks: the center of the femoral head, entry point of the femoral canal, trochlear groove, and medial and lateral femoral epicondyles. The axis of the posterior condyle (using a specific posterior condyle “digitizer”) was then preliminarily set at 0° after consideration of potential posterior cartilage or bone loss. We then proceed with landmarking the distal surfaces of the medial and lateral femoral condyles, considering again that the normal thickness of the human knee cartilage was about 2 mm. During landmarking, we set the level of bone resection in a way that the articular surface of the components matched the native cartilage. Once the femoral landmarking was completed, we addressed the tibia. The medial and lateral malleoli, the medial third of the tibial tubercle, the tibial canal entry point, the posterior cruciate ligament insertion point, and the center points of the medial and lateral tibial plateau were all landmarked in sequence. After this, the knee landmarking was completed.

**Fig. 2:**
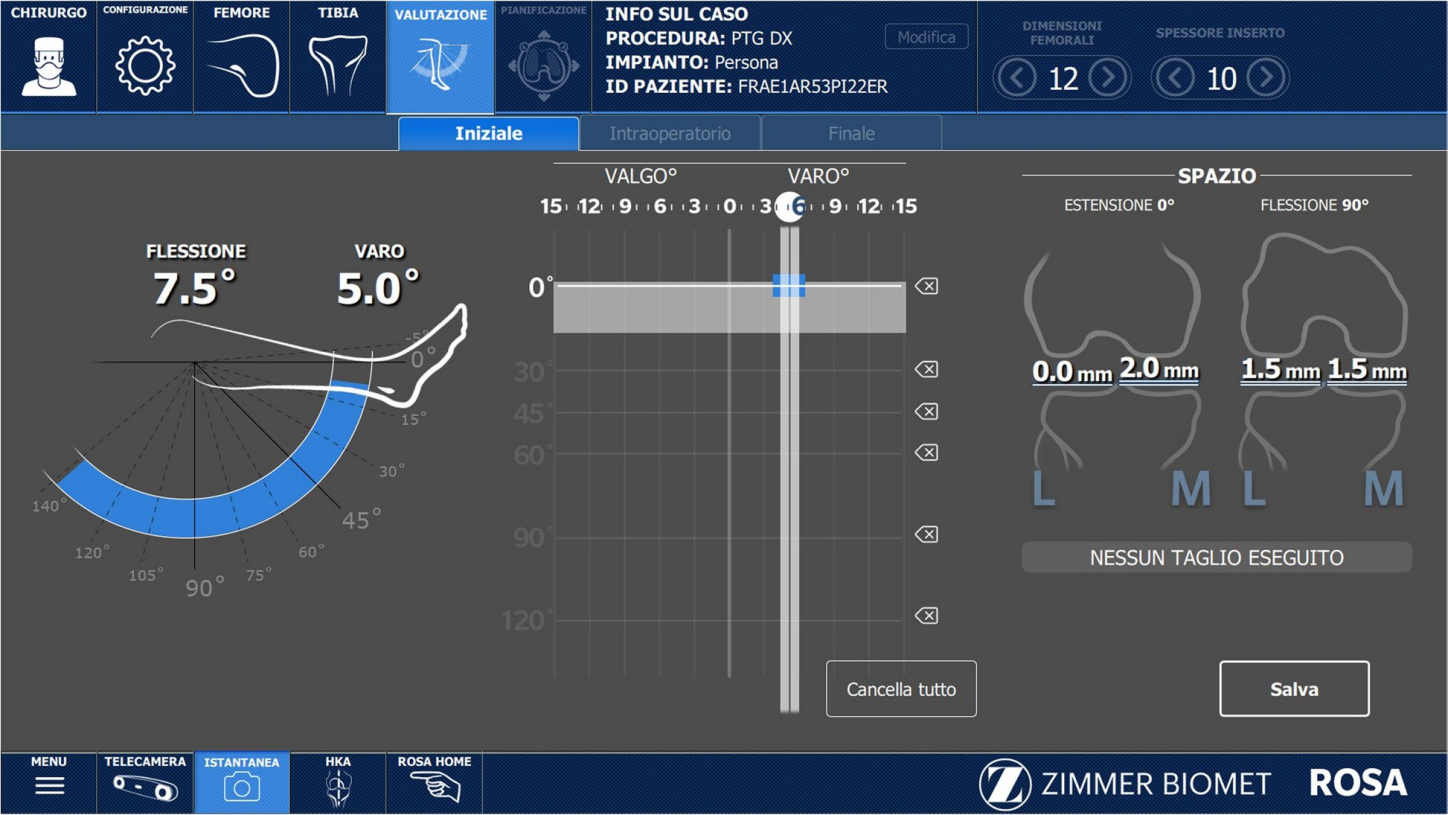
Right knee. Evaluation of the right knee preoperative alignment and preoperative flexion contracture after landmarking is completed. The knee has a 7.5° flexion contracture and an overall alignment in 5° of varus with respect to the Hip Knee Ankle (HKA) axis

**Fig. 3:**
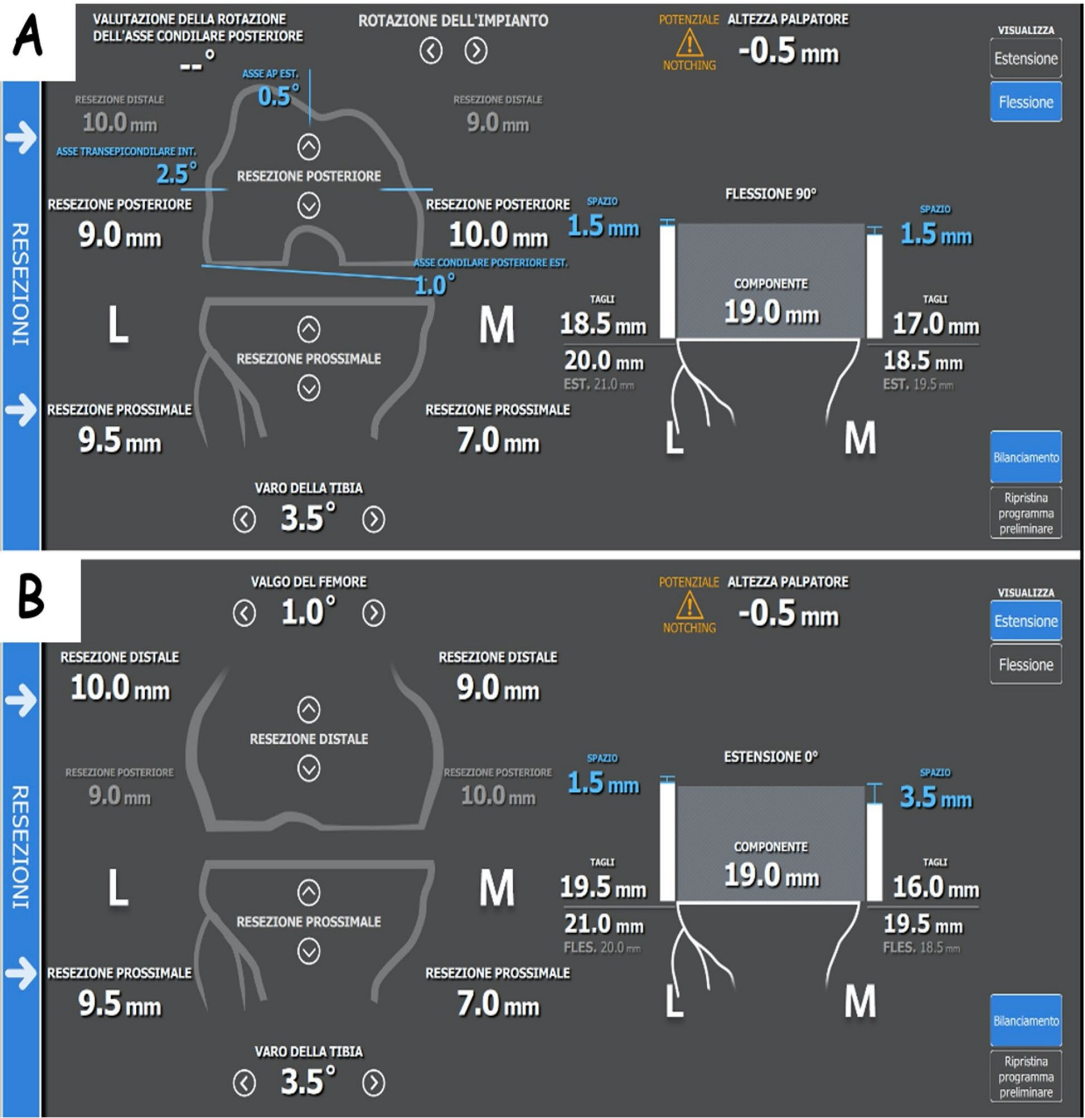
**A** Right knee. The first step of this technique requires the determination of the flexion gap, which is planned to be slightly tighter in the medial compartment compared to the lateral. In this case, the medial side flexion gap was planned to be 1.5 mm tighter than the lateral, thanks to the slightly asymmetric posterior femur and proximal tibia bone resections. Here, the external rotation of the femoral component is set at 1° with respect to the posterior condylar axis: this differs significantly from standard 3° which is recommended by the mechanical alignment technique. **B** Intraoperative planning of the extension gap. Like the flexion gap, the extension gap is planned to be slightly tighter in the medial compartment compared to the lateral one. In this case, the medial side flexion gap was also planned to be 1.5 mm tighter than the lateral, thanks to the slightly asymmetric bone resections. The postoperative Medial Proximal Tibial Angle is planned to be in a 3.5° of varus with respect to the Hip-Knee-Ankle axis

We then evaluated the knee’s range of motion and alignment (Fig. 2). Prior to osteophyte removal, we applied a robot-assisted varus/valgus stress between 0° and 120° to quantify patient-specific intercompartmental laxity and the possibility of deformity correction in both extension and in flexion. The goal of our “easy” rKA technique was to facilitate the reproduction of natural knee kinematics, as determined by multiple gait analysis studies in non-arthritic knees [3, 11, 12]. We planned for a slight asymmetry (2 mm) in the extension, mid-flexion, and full flexion gaps; we believed that adding a medially-congruent TKA design (Persona MC, Zimmer Biomet, Warsaw, IN, USA) was going to facilitate the achievement of this goal too. The bone cuts strategy focuses on determining the correct flexion gap as the initial step (Fig. 3), different from other robotic-assisted and computer navigation techniques. The Persona MC (Zimmer Biomet, Warsaw, IN, USA) knee system requires 19 mm of space to accommodate the components’ thicknesses. Femoral rotation, tibial slope, and anterior and posterior femoral bone cuts were determined during the intraoperative planning to match this 19 mm requirement. As shown in the flexion gap screenshot (Fig. 3), the thickness of the bone cuts differed significantly from the traditional unrestricted kinematic alignment technique, which aims for symmetric bone cuts and symmetric flexion/extension gaps. Still, the traditional mechanical alignment landmarks (trans-epicondylar axis, posterior condylar axis, etc.) were used as a reference to recreate this slightly asymmetric flexion gap and not as a dogma to be respected at any cost. To that end, a slight internal rotation of the femoral component with respect to the posterior condylar axis (traditionally set at 3° when a mechanical alignment approach is followed) was obtained during the execution of this technique (Fig. 3). Only after the slightly asymmetric flexion gap was successfully determined, attention turned to the extension gap which also reflected the final alignment of the knee (Fig. 3). In this phase, the surgeon’s focus was on confirming that the LDFA and the MPTA fell inside 0° ± 5° with respect to the Hip-Knee-Ankle (HKA) axis, which is the case for the majority (65% of cases, according to Almaawi et al. [13]) of knee patho-anatomies undergoing primary TKA. In extreme deformities where the LDFA and the MPTA fall outside 0° ± 5° with respect to the HKA axis, the current authors recommend, differently from other kinematic alignment principles, performing personalized soft tissue releases to obtain a satisfactory final, slightly asymmetric, balance in extension and in flexion. Once all these principles were applied, the final alignment of a preoperatively varus knee was postoperative varus alignment (Fig. 3) with a slight varus (1°) with respect to the HKA axis. Particular attention was paid to the tibial slope, which is, in the current authors’ opinion, design-specific. The MC system has been shown to achieve great stability even in a weight-bearing, flexion scenario [14]. In this fact, we recommend sacrificing the posterior cruciate ligament and setting the tibial slope between 5° and 7° without fear of postoperative flexion instability, even in the valgus knee.

**Fig. 4:**
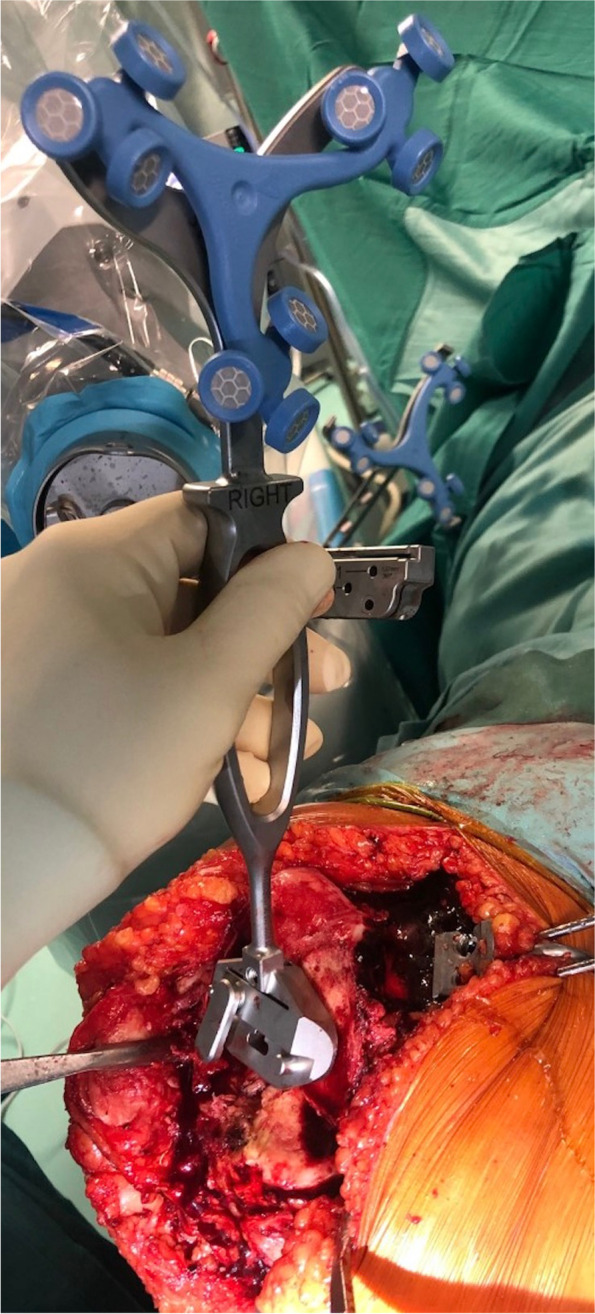
Right knee. Use of the wireless verification tool on the femur to ensure that the Distal Lateral Flexion Angle ended at 0° ± 5° respected to the Hip-Knee-Ankle (HKA) axis

**Fig. 5:**
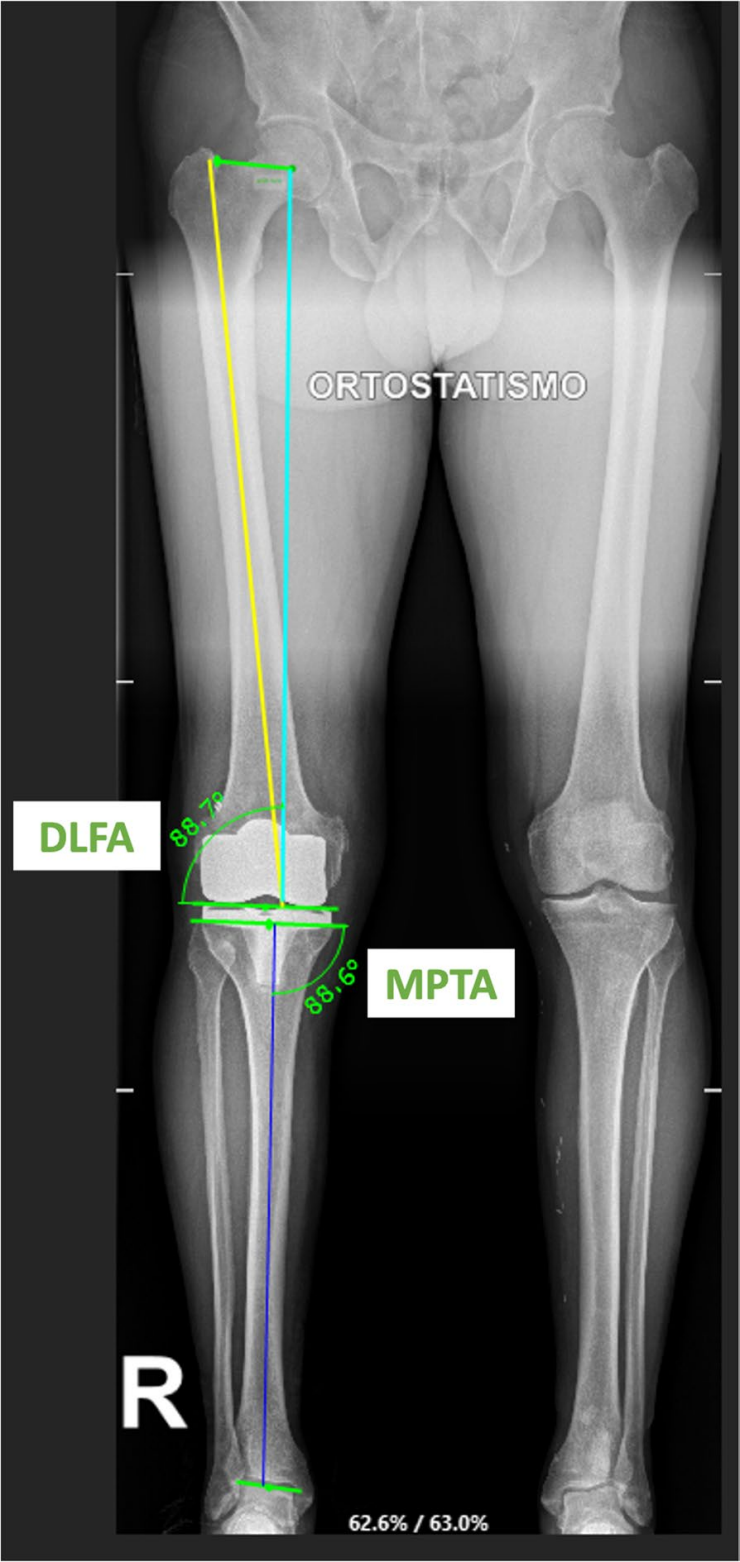
A 69-year-old male. Right knee postoperative radiographs. Right lower extremity long film with an overall varus alignment and LDFA/MPTA < 5° respect to HKA; LDFA = 88.7°; MPTA: 88.6°. The overall alignment respected to the HKA is 1.26° varus. The intra-operatively planned HKA alignment was 1° varus (LDFA: lateral distal femoral angle; MPTA: medial proximal tibial angle; HKA: Hip-Knee-Ankle axis)

We performed resections in the following sequence: distal femur, rotational alignment femoral component, femoral chamfer cuts, proximal tibia, and tibial finishing last. The robotic arm was connected in a collaborative mode to the intraoperative planning software to verify all resection thicknesses before the final positioning of the cutting blocks. The cutting blocks were not secured during distal femoral and proximal tibia resection to allow the jig to adjust to any involuntary motion of the patient’s leg. However, the femoral “four-in-one” and tibial finishing jigs were secured to the joint surfaces with headed pins before final resections. Once the distal femur/proximal tibia bone cuts were completed, we utilized the wireless verification tool (Fig. 4) to ensure that the LDFA and MPTA ended at 0° ± 5° with respect to the HKA axis, as this represents the alignment goals of this “easily-restricted” kinematic alignment technique. Once the trial components were in place, we meticulously checked the knee range of motion, the patellofemoral tracking, and the mediolateral stability of the joint between full extension and maximum flexion, aiming for slight gap asymmetry during the entire motion. Historically, this asymmetry has been evaluated according to the surgeons’ experience, in a very subjective way; the use of this robotic system allows for accurate quantification of the desired, constitutional, greater laxity of the lateral compartment, from full extension to high grades of knee flexion. In a previous gait-analysis study [11], the current authors showed that the MC bearing represents an ideal solution to assist in the recreation of the natural, close-to-normal, knee kinematics. After this final intraoperative robot-assisted evaluation, the trial components were removed together with the trackers and pins. The knee joint was then prepared for the final cementation of the components, positioning of the final MC insert, and closure of the capsule, soft tissue, and skin in a standard fashion. Standard postoperative AP and lateral radiographs were obtained in the recovery room and standing long-leg radiographs were obtained 6 weeks after the index procedure (Fig. 5). At that time, the LDFA and MPTA were measured and compared with the intraoperative, robotic-assisted plan.

## Discussion and conclusion

In this note, we presented a simplified, robot-assisted, restricted kinematic alignment total knee arthroplasty surgical technique. This technique simplifies the recognition of pre-defined alignment boundaries integrating the kinematic principle of slight extension and flexion gaps asymmetry to improve the stability of the knee, especially during the swing phase of gait.

Over the last 30 years at the authors’ institution, multiple kinematic parameters during normal gait (knee flexion and extension angle, adduction-abduction angle, internal and external tibial rotation, peak knee flexion moment, peak knee internal rotation moment, etc.), have been studied in non-arthritic and arthritic patients with varus deformity. The authors’ previous reports [3, 11] highlighted two main findings:

1. Both in the natural knee and in the varus osteoarthritic knee, there exists a significant difference in load between the medial and lateral compartments, with the medial side experiencing 2–3 times increased load compared to the lateral one [3]. This difference is responsible for a slight opening of the lateral side of the knee (in both flexion and extension) when the knee is tested in a non-weight-bearing situation;
2. There is also a robust difference in the inter-compartmental knee kinematics during the stance phase of gait (center of rotation in the lateral compartment) compared to the swing phase of gait (center of rotation in the medial compartment) due to anterior cruciate ligament tension [12].

Our previous study [11] comparing posterior-stabilized TKA patients and healthy controls found several differences in knee kinematics. Specifically, the posterior-stabilized knees lacked full extension at heel-strike, had less peak internal rotation during stance, required an increase in hamstring muscle activation to counteract the “paradoxical motion” typical of posterior stabilized TKA designs, and showed fewer late stance peak extension moments compared to controls. These findings suggest that posterior-stabilized TKA designs may not fully restore normal knee kinematics during gait and may require modifications to improve outcomes.

Inspired by our gait analysis data, we developed a deep interest in alternative alignment strategies during TKA in search of more normal knee kinematics. Multiple authors from different countries recently challenged the dogma that mechanical alignment represents the gold standard for TKA. The real “godfather” of an alternative alignment strategy was Dr. Steve Howell, who introduced [5] the “kinematic alignment” (KA) principles. According to Howell et al. [5], kinematic alignment resurfaces the knee to restore pre-arthritic anatomy while minimizing soft tissue releases. On this path, other authors recently proposed several alignment strategies as alternatives to mechanical alignment: the “restricted KA” (rKA) by Vendittoli et al. [6], the “inverse KA” by Winnock de Grave et al. [8], the “personalized alignment” by Lustig et al. [7] and the “functional alignment” by Chang et al. [15]. Classic kinematic alignment favors preservation of the native soft tissue laxity through pure resurfacing of the knee joint [5], “restricted” kinematic alignment defines a “safe zone” for TKA alignment [6], while “inverse” kinematic alignment [8] and functional alignment [9, 15] aim for equal, isometric gaps in extension and flexion. We proposed the use of an imageless robotic system to intraoperatively perform a dynamic gap measurement during passive ROM, aiming for the establishment of mild (1–2 mm) gap imbalances within restricted Kinematic Alignment boundaries: the current technique differs from other personalized alignment techniques because it is based on the initial determination of a slightly asymmetric flexion gap, followed by the reproduction of a slightly asymmetric extension gap (Table 1). The current and other authors [11] recently showed that the natural knee is characterized by a constant lateral laxity, especially during knee flexion: interestingly, it has been shown that this natural, flexion gap laxity is also associated with improved clinical outcomes [16]. We strongly believe that looking only at the static HKA axis as a determinant for limb alignment, as proposed by many of the above techniques, will not necessarily increase the satisfaction rate in patients. Many surgeons [3, 11] demonstrated the coronal alignment measured on a weight-bearing X-ray limits accurate kinematics prediction in TKA patients, particularly during the swing phase of gait.

**Table 1:** Differences between commonly used personalized Total Knee Arthroplasty techniques

**Technique**	**KA **[5]	**rKA **[6]	**Easy rKA**	**iKA **[8]	**FA **[9, 16]
Planning surgical steps	Femur first (ext. gap first)	Femur first (ext. gap first)	Femur first (flex. gap first)	Tibia first (ext. gap first)	Femur First (ext. gap first)
Knee balancing drivers	Tension native ligaments	Tension native ligaments	Slight asymmetry gaps (1–2 mm)	Ligaments Isometry	Ligaments Isometry
Tools	Caliper (manual)	Robotics recommended	Robotics mandatory	Robotics	Robotics
Femoral distal cut	Wear determined	Parallel to distal femoral joint line (HKA ± 3°)	DLFA ± 5°	Guided by tibial cut	Parallel to distal femoral joint line (Target: 0°–5°)
Femoral posterior cut	Parallel PCA	Parallel PCA	Slight asymmetry flex gap	Guided by tibial cut	Surgical TEA
Tibial coronal cut	Symmetric (base of ACL spine)	Parallel to tibia joint line (HKA ± 3°)	MPTA ± 5°	Safe zone: 6°varus/2°valgus	Perpendicular tibial MA
Tibial slope	Parallel medial plateau slope	Parallel lateral plateau slope	5°–7° (MC insert)	Parallel medial plateau slope	Parallel medial plateau slope (0°–3°)
Tibial rotation	Parallel axis lateral plateau	Matching with femur in extension	Maximum coverage tibial plateau	Parallel axis lateral plateau	Target: Akagi’s line
Soft tissue releases	Never	Only for extreme preoperative alignments	Occasionally to correct severe deformities	Sometime	Rarely

Details of different Personalized Total Knee Arthroplasty Techniques: *KA* Kinematic Alignment, *rKA* Restricted Kinematic Alignment, *iKA* Inverse Kinematic Alignment, *FA* Functional Alignment, *ext.* extension, *flex.* flexion, *HKA* Hip/Knee/Ankle axis, *DLFA* Distal Lateral Femoral Angle, *PCA* Posterior Condylar Axis, *TEA* Trans-epicondylar axis, *MPTA* Medial Proximal Tibial Angle, *MA* mechanical axis, *MC* Medially Congruent

The easily-restricted kinematic alignment technique described in this note utilizes a robotic system to aid in the reproduction of patient-specific constitutional alignment (varus or valgus), maintenance of existing soft tissue tension, creation of a slightly asymmetric flexion (first) and extension (second) gaps to favor the posterolateral rollback of the femur during the swing phase of gait. This was all done within acceptable alignment boundaries as proposed by Vendittoli et al. in their original technique [6]. Accuracy in identifying the native alignment and soft tissue tension is critical to formulating a preoperative plan focused on reproducing natural knee kinematics as closely as possible. A major advantage of an imageless robotic system is that it eliminates the need for preoperative radiological planning (anteroposterior weight-bearing, lateral weight-bearing, full leg radiographs and patella sunrise view), as the system can easily identify the alignment boundaries based on the patient’s individual anatomy. Another advantage of this technique is that it allows for a precise setting of 0° ± 5° for the LDFA and the MPTA with respect to the HKA axis. Vendittoli et al.’s work [6] has been instrumental to the understanding of arthritic knee patho-anatomy, showing a mean LDFA value of 2.7° valgus and a mean MPTA value of 2.9° varus in 4,884 lower limb CT-scans of patients scheduled for TKA. While personalized alignment techniques and robotics can improve the accuracy of implant placement and soft tissue balancing, current implant designs may not fully replicate normal knee kinematics.

We presented a personalized, robot-assisted, simplified, TKA surgical technique which has the goal of reproducing closer-to-normal medial pivot kinematics. The three fundamental elements of this technique are represented by “safe to identify” alignment boundaries, the use of a modern, imageless robotic system, and the use of a medially congruent insert which has shown satisfactory clinical outcomes. The use of easily-identifiable alignment boundaries can help ensure that the implant is positioned within acceptable alignment boundaries, reducing the risk of implant loosening or migration. In addition, the use of robotics allows for real-time assessment of soft tissue tension during the range of motion, which can help to ensure that the implant is appropriately balanced and stable throughout the full range of motion. This differs from the traditional mechanical alignment approach, which focuses on static coronal plane alignment without accounting for soft tissue tension during the range of motion. By incorporating both alignment boundaries and dynamic soft tissue tension assessment, the personalized, robot-assisted technique may offer improved outcomes compared to traditional mechanical alignment techniques.

## Data Availability

The datasets used and/or analyzed during the current study are available from the corresponding author on reasonable request.

## Abbreviations

TKA: Total Knee Arthroplasty
HKA: Hip-Knee-Ankle axis
KA: Kinematic Alignment
rKA: Restricted Kinematic Alignment
LDFA: Lateral Distal Femoral Angle
MPTA: Medial Proximal Tibial Angle
MC: Medially Congruent
ROM: Range of Motion
